# Synergistic Activity between Two Antifungal Proteins, the Plant Defensin NaD1 and the Bovine Pancreatic Trypsin Inhibitor

**DOI:** 10.1128/mSphere.00390-17

**Published:** 2017-10-18

**Authors:** Mark R. Bleackley, Charlotte S. Dawson, James A. McKenna, Pedro Quimbar, Brigitte M. E. Hayes, Nicole L. van der Weerden, Marilyn A. Anderson

**Affiliations:** Department of Biochemistry and Genetics, La Trobe Institute for Molecular Science, La Trobe University, Bundoora, Victoria, Australia; Carnegie Mellon University

**Keywords:** antifungal peptides, fungi, defensins

## Abstract

Defensins are a large family of small, cationic, cysteine-rich proteins that are part of the defense arsenal that plants use for protection against potentially damaging fungal infections. The plant defensin NaD1 from *Nicotiana alata* is a potent antifungal protein that inhibits growth and kills a variety of fungal pathogens that affect both plant and animal (human) hosts. Some serine protease inhibitors have also been reported to be antifungal molecules, while others have no inhibitory activity against fungi. Here we describe the synergistic activity of the plant defensin NaD1 with a selection of serine protease inhibitors against the plant pathogens *Fusarium graminearum* and *Colletotrichum graminicola* and the animal pathogen *Candida albicans*. The synergistic activity was not related to the protease inhibitory activity of these molecules but may arise from activation of fungal stress response pathways. The bovine pancreatic trypsin inhibitor (BPTI) displayed the most synergy with NaD1. BPTI also acted synergistically with several other antifungal molecules. The observation that NaD1 acts synergistically with protease inhibitors provides the foundation for the design of transgenic plants with improved resistance to fungal disease. It also supports the possibility of naturally occurring accessory factors that function to enhance the activity of innate immunity peptides in biological systems.

**IMPORTANCE** This work describes the increased activity of a natural antifungal peptide in the presence of another antifungal peptide from a different family. This is termed antifungal synergy. Synergy is important for decreasing the amount of antifungal molecule needed to control the disease. Traditionally, naturally occurring antifungal molecules are assayed in isolation. Identification of synergistic interactions between antifungal peptides means that their activities in a complex biological system are likely to be different from what we observe when examining them individually. This study identified synergy between an antifungal peptide and a group of peptides that do not affect fungal growth *in vitro*. This provides the foundation for generation of transgenic plants with increased resistance to fungal disease and identification of antifungal accessory factors that enhance the activity of innate immune molecules but do not have an antifungal effect on their own.

## Abstract (precis)

This work describes the increased activity of a natural antifungal peptide in the presence of another antifungal peptide from a different family. This is termed antifungal synergy. Synergy is important for decreasing the amount of antifungal molecule needed to control the disease. Traditionally, naturally occurring antifungal molecules are assayed in isolation. Identification of synergistic interactions between antifungal peptides means that their activities in a complex biological system are likely to be different from what we observe when examining them individually. This study identified synergy between an antifungal peptide and a group of peptides that do not affect fungal growth *in vitro*. This provides the foundation for generation of transgenic plants with increased resistance to fungal disease and identification of antifungal accessory factors that enhance the activity of innate immune molecules but do not have an antifungal effect on their own.

## INTRODUCTION

Fungi cause serious disease in both agriculture and medicine. It has been estimated that the amount of food lost each year due to fungal disease could feed 8.5% of the world’s population and that coincidental epidemics in major crops have the potential to decrease food production to less than 40% of current levels (1). Fungal infections in humans range from superficial infections on skin and mucous membranes that are relatively easy to treat to invasive infections that have mortality rates that often exceed 50% (2). Immunocompromised individuals are particularly susceptible to life-threatening systemic fungal infections (3). Control of fungal pathogens has been traditionally accomplished through the use of small-molecule antifungal agents that exploit either the fungus-specific sterol ergosterol or the unique composition of the fungal cell wall (4). Resistance to established antifungal treatments (5, 6) has created a need for new methods for control of fungal pathogens in both agriculture and medicine.

Synergistic activity between two or more different molecules has become an area of great interest in the control of microbial pathogens. A synergistic interaction between antimicrobial agents occurs when growth inhibition by a combination of molecules is greater than that predicted from the additive effects of the two molecules based on their activities in isolation (7). Antimicrobial synergy decreases the amount of drug required to clear an infection and in some cases allows drugs to contact previously inaccessible targets (8). In theory, combinations of antimicrobials also provide a more significant barrier to development of resistance because multiple independent mutations are required for resistance to multiple drugs (8). However, *in vitro* experiments on the evolution of resistance have revealed that resistance can develop more rapidly to drugs in some synergistic combinations than when the drugs are being used separately, particularly when the dosages are near the MIC (9). Nonetheless, synergy remains a useful tool for design of new treatment regimens for microbial pathogens.

Naturally occurring antifungal peptides (AFPs) are an attractive set of molecules on which to focus efforts for the development of new strategies for control of fungal diseases. AFPs are found throughout all kingdoms of life and have a diverse range of structures and mechanisms of action (10). One group of AFPs that has received significant focus are the plant defensins that are highly variable in sequence apart from the cysteine residues that dictate the conserved defensin fold (11). The mechanisms of action of only a few members of this large family have been studied in detail, and those with highly divergent sequences act via different mechanisms (12). A prevailing trend in the investigation of plant defensins and other antifungal peptides is to assess their ability to act synergistically with established antifungal drugs of the polyene, azole, and/or echinocandin family. For example, HsAFP1 (*Heuchera sanguinea* antifungal peptide 1), a defensin from *Heuchera sanguinea*, acts synergistically with both amphotericin B and caspofungin against *Candida albicans* (13). The mechanism underlying this synergy is not yet known. The radish defensins RsAFP1 (*Raphanus raphanistrum* subsp. *sativus* AFP1) and RsAFP2 also act synergistically with caspofungin against *C. albicans* (14), but again, no mechanism for this interaction has been proposed. A capsicum thionin, CaThi (*Capsicum annuum* thionin), which belongs to a family of AFPs with many similarities with defensins, works synergistically with fluconazole against several *Candida* species (15). CaThi is proposed to induce changes in the fungal plasma membrane that enhance the ability of fluconazole to traverse the membrane and access the intracellular target.

Synergy between plant defensins and small-molecule antifungal agents has applications where antifungal agents are applied exogenously. However, one of the advantages of plant defensins is that they are encoded by genes and can be used to generate transgenic plants with increased resistance to fungal disease (16–18). Thus, it is important that the potential for synergistic antifungal activity between two gene-encoded AFPs with different mechanisms of action be investigated.

Protease inhibitors (PIs) are produced by plants and have a major role in defense against herbivorous insect pests (19, 20). Fungi produce a variety of proteases that function in various physiological processes (21). Antifungal activities have also been reported for some plant protease inhibitors (22–24) as well as the bovine pancreatic trypsin inhibitor (BPTI) (25). Some of these antifungal protease inhibitors act by inhibiting proteases that are essential for fungal viability, while others have nonprotease targets (22–25).

We have identified synergistic antifungal activity between two antifungal peptides from different protein families. These antifungal peptides are NaD1, a member of the plant defensin family, and BPTI, a Kunitz-type serine protease inhibitor. Synergy was assessed on the plant pathogens, *Fusarium graminearum* and *Colletotrichum graminicola*, as well as the human pathogen *C. albicans*. Non-Kunitz family protease inhibitors also acted synergistically with NaD1, but not to the same level as with BPTI. Investigation of the mechanism of synergy between NaD1 and BPTI revealed that the protease inhibitory activity of BPTI was not required for synergy and that BPTI also acted synergistically with other nondefensin antifungals. There is evidence to support a role for the high-osmolarity glycerol (HOG) and cell wall integrity mitogen-activated protein (MAP) kinase cascades in the synergy between BPTI and NaD1. Identification of the synergistic activity of these two classes of antifungal peptides provides new avenues for the development and use of antifungal peptides for the control of fungal disease.

## RESULTS

### Synergy between NaD1 and serine protease inhibitors.

*Fusarium graminearum*, *Colletotrichum graminicola*, and *Candida albicans* were assessed for susceptibility to a set of serine protease inhibitors: bovine pancreatic trypsin inhibitor (BPTI), lima bean trypsin inhibitor (LBTI), Bowman Birk inhibitor from *Glycine max* (soybean) (BBI) and chymotrypsin inhibitor from barley (CI-1B) both alone and in combination with the plant defensin NaD1. Standard checkerboard assays were employed in the first experiments. In the absence of NaD1, none of these protease inhibitors had a substantial impact on the growth of any of the fungal species tested at concentrations up to 10 µM. The MICs for these protease inhibitors were arbitrarily set at 20 µM, and the minimum fractional inhibitory concentration (FIC) value for each combination was determined (Table 1). BPTI had the lowest FIC value of any of the protease inhibitors against each of the fungal species. The FIC value was below the synergy cutoff of 0.5 for *C. albicans* (0.45 ± 0.05). The FIC values for NaD1 and BPTI against *C. graminicola* and *F. graminearum* were just above the synergy cutoff with values of 0.63 ± 0.12 and 0.56 ± 0.02, respectively.

**TABLE 1:** Minimum FIC values for combinations of serine protease inhibitors with the plant defensin NaD1 against three fungal species

Serine protease inhibitor	Minimum FIC value^a^ for serine protease inhibitor with NaD1 against:		
	*F. graminearum*	*C. graminicola*	*C. albicans*
BPTI	0.54 ± 0.02	0.63 ± 0.12	0.45 ± 0.05
LBTI	0.88 ± 0.30	1 ± 0	0.73 ± 0.15
BBI	1 ± 0	1 ± 0	0.81 ± 0.16
CI-1B	0.73 ± 0.31	1 ± 0	0.83 ± 0.20

a Values are averages ± 95% confidence intervals from at least three independent replicates.

**FIG 1:**
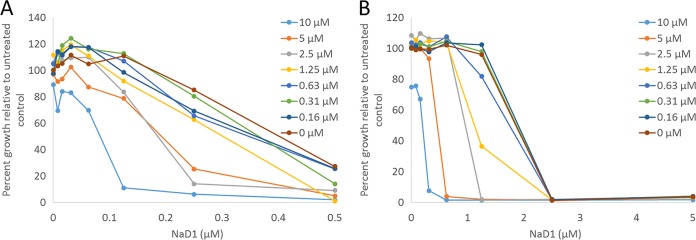
Synergy between NaD1 and BPTI against *F. graminearum* and *C. albicans*. Growth inhibition of *F. graminearum* (A) and *C. albicans* (B) by NaD1 occurs at lower concentrations as the concentration of BPTI (colored lines) increases. Each graph shows data from a single checkerboard assay, and the results shown are representative of three independent experiments.

In the presence of subinhibitory concentrations of NaD1, the protease inhibitors caused a concentration-dependent inhibition of fungal growth (Fig. 1). As the MICs for the protease inhibitors had been arbitrarily set at 20 µM, the FIC calculation was likely to underestimate the synergistic interaction between protease inhibitors and NaD1. Thus, a second method to calculate synergy with NaD1 was also used for these combinations. In this method, synergy is ascribed when the inhibition caused by a combination of two molecules exceeds the inhibitory effect that would be predicted by the inhibitory effect of the two molecules independently. This difference, the inhibition difference (ID), was calculated using Limpel’s formula. Synergistic combinations of defensin and protease inhibitor (PI) were defined as those with an ID value greater than zero. BPTI acted synergistically with NaD1 against all fungal species tested and yielded the highest ID values (Table 2). *F. graminearum* was the most susceptible of the fungi tested to the synergistic activity of NaD1 with the widest range of PIs, as all combinations of PIs with NaD1 yielded a synergistic growth inhibitory effect. The magnitude of synergy on *F. graminearum* varied from an ID of 28.7 ± 5.9 for LBTI to 86.9 ± 6.5 for BPTI. *C. albicans* was similarly susceptible to the synergistic activity of BPTI with a synergy value of 80.1 ± 7.5 and was the most sensitive of the fungi tested to the synergistic activity of NaD1 with LBTI with an ID of 64.8 ± 6.5. Minimal to no synergy was observed for the combination of NaD1 with BBI or wild-type barley chymotrypsin inhibitor CI-1B against *C. albicans*. *C. graminicola* was the least sensitive of the fungi tested to enhancement of growth inhibition by NaD1 in the presence of PIs. The only combination with strong synergy against this fungus was NaD1 and BPTI with a synergy value of 76.5 ± 2.9.

**TABLE 2:** Maximum synergy values of serine protease inhibitors with NaD1 calculated using Limpel’s formula^a^

Serine protease inhibitor	*F*. *graminearum*			*C*. *graminicola*			*C. albicans*		
	Ie	Io	ID	Ie	Io	ID	Ie	Io	ID
BPTI	4.7 ± 4.4	91.6 ± 3.5	86.9 ± 6.5	19.6 ± 1.7	96.1 ± 2.4	76.5 ± 2.9	11.4 ± 6.7	91.5 ± 7.7	80.1 ± 7.5
LBTI	8.3 ± 4.3	37.0 ± 7.7	28.7 ± 5.9	11.0 ± 6.3	13.5 ± 2.9	2.5 ± 6	10.9 ± 3.3	75.7 ± 15.0	64.8 ± 6.5
BBI	8.2 ± 4.1	75.2 ± 4.0	67 ± 5	6.36 ± 8.3	11.4 ± 5.2	5.04 ± 8	3.3 ± 3.5	14.9 ± 4.5	11.6 ± 4.7
CI-1B	4.3 ± 7.4	89.7 ± 9.0	85.4 ± 16.8	15.5 ± 0.02	16.6 ± 3.2	1.1 ± 2	4.5 ± 3.5	0.76 ± 1.8	−3.74 ± 3.8

a Ie is the expected growth inhibition, Io is the observed growth inhibition, and ID is the difference in observed and expected inhibition levels. Data are averages from three independent experiments. Ie and Io data are means ± standard deviations for at least three replicates. ID data are means ± 95% confidence intervals.

As *F. graminearum* responded to the broadest range of PIs in combination with NaD1, the synergy assays were extended to include At2g38870, NaPin1a, and NaCys1 (Table 3). These protease inhibitors exhibited different levels of synergy with NaD1 with At2g38870 being the most potent with an ID of 75.4 ± 3.7, followed by NaCys1 with an ID of 51 ± 11.3 and NaPin1a with an ID of 32 ± 12.6. Small-molecule serine protease inhibitors 4-(2-aminoethyl)benzenesulfonyl fluoride (AEBSF) and benzamidine were also assessed for synergistic activity with NaD1 against *F. graminearum* (Table 4). No synergy was observed for either of the small-molecule protease inhibitors.

**TABLE 3:** Synergy between other protease inhibitors and NaD1 against *F. graminearum*^a^

Protease inhibitor	Description	Ie	Io	ID
At2g38870	*Arabidopsis* type I inhibitor	8.69 ± 0.5	84.1 ± 9.1	75.41 ± 3.7
NaPin1A	*N. alata* type I inhibitor	12.8 ± 13.1	44.8 ± 12.7	32 ± 12.6
NaCys1	*N. alata* cystatin	25.5 ± 10.1	76.5 ± 17.5	51 ± 11.3

a Ie and Io values are means ± standard deviations for three replicates. ID values are means ± 95% confidence intervals.

**TABLE 4:** Maximum synergy values for combinations of NaD1 and small-molecule protease inhibitors against *F. graminearum* calculated using Limpel’s formula^a^

Protease inhibitor	Description	Ie	Io	ID
AEBSF	Serine protease inhibitor	33.0 ± 6	47.0 ± 3	14 ± 5.9
Benzamidine	Trypsin inhibitor	17.3 ± 13.7	7.1 ± 6.8	−10.2 ± 11.9

a Ie is the expected growth inhibition, Io is the observed growth inhibition, and ID is the difference in observed and expected inhibition levels. Data are averages from three independent experiments. Ie and Io values are means ± standard deviations for at least three replicates. ID values are means ± 95% confidence intervals.

**FIG 2:**
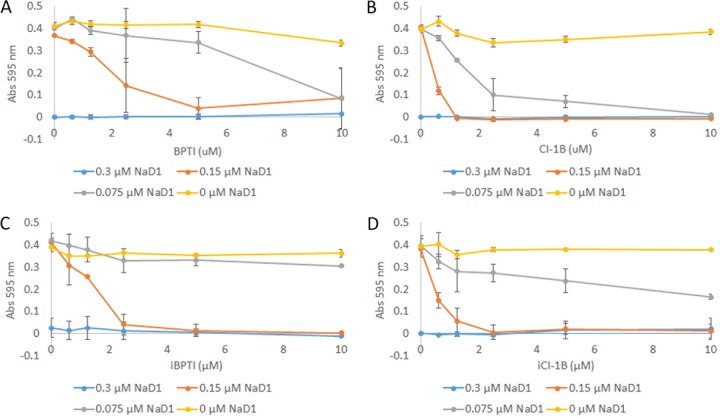
Synergy between NaD1 and inactive protease inhibitor variants against *F. graminearum*. Synergy was observed between NaD1 and inactive variants of BPTI and CI-1B that do not inhibit their target proteases due to variations in residues that are crucial for protease inhibition. The levels of synergy were similar for the active (A and B) and inactive (C and D) variants. The maximum ID values for the inactive variants are 91.9 ± 12.7 and 92.5 ± 3.0 for iBPTI and iCI-1B, respectively (these values are averages of three independent experiments ± 95% confidence intervals). Data presented in graphs are representative of three independent experiments. Error bars are standard deviations of technical duplicates. Abs 595 nm, absorbance at 595 nm.

The lack of synergy between small-molecule serine protease inhibitors and NaD1 led to the hypothesis that inhibition of proteases by the proteinaceous protease inhibitors may not be the cause of synergy. To test this hypothesis, variants of BPTI and CI-1B with substitutions of reactive-site residues crucial for inhibition of target proteases (iBPTI K15A R17A [inactive variant of BPTI with K15A and R17A substitutions] and iCI-1B L63A [inactive variant of CI-1B with L63A substitution]) were assessed for synergy with NaD1 against *F. graminearum*. Synergy between the inactive protease inhibitors and NaD1 was not significantly different (95% confidence intervals overlap) from the wild-type protease inhibitors with ID values of 91.9 ± 12.7 and 92.5 ± 3.0 for iBPTI and iCI-1B, respectively (Fig. 2).

### Inhibition of trypsin and chymotrypsin.

The molecules assessed in the synergy assays were also evaluated in protease inhibition assays using trypsin and chymotrypsin. Cleavage of an appropriate fluorescent substrate by the protease was monitored kinetically in the presence of a range of concentrations of inhibitor. Data were fitted to Morrison’s equation to determine the *K*_*i*_ values for each molecule for both trypsin and chymotrypsin (Table 5). BPTI inhibited trypsin with the lowest *K*_*i*_. BBI and LBTI had *K*_*i*_s approximately 10-fold higher than the *K*_*i*_ for BPTI and trypsin, and CI-1B did not inhibit trypsin over the concentrations that were assessed. All of the PIs inhibited chymotrypsin with a *K*_*i*_ ranking of BBI < LBTI < BPTI < CI-1B. NaD1 did not inhibit either trypsin or chymotrypsin. These protease inhibition assays were also used to confirm the lack of inhibitory activity for iBPTI and iCI-1B against their target proteases.

**TABLE 5:** Inhibitory activities of protease inhibitors against bovine trypsin and chymotrypsin

Protease inhibitor	*K*_*i*_ (nM)^a^ of protease inhibitor against:	
	Trypsin	Chymotrypsin
BPTI	1 × 10^−2^ ± 5 × 10^−3^	6.3 ± 3.7
CI-1B	>1,000	175.5 ± 6.4
BBI	0.77 ± 0.24	0.92 ± 0.45
LBTI	0.16 ± 0.064	1.12 ± 0.60
iBPTI	>1,000	NA
iCI-1B	NA	>1,000
NaD1	>1,000	>1,000

a Values are averages ± standard deviations from three independent experiments. NA, not available.

### Effects of protease inhibitors on growth of *F. graminearum*.

**FIG 3:**
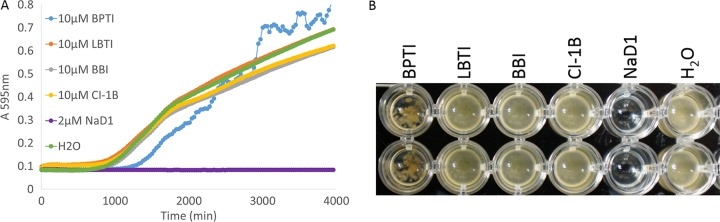
Growth of *F. graminearum* in the presence of serine protease inhibitors. (A) Absorbance at 595 nm of an *F. graminearum* culture grown in the presence of PIs or NaD1 over time. NaD1 (2 µM) completely inhibited the growth of *F. graminearum* over the course of 66 h. BPTI (10 µM) inhibited fungal growth for a short time. Fungi treated with CI-1B (10 µM), LBTI (10 µM), and BBI (10 µM) displayed growth patterns similar to that of fungi treated with the H_2_O control. Data are representative of three independent experiments. (B) Image of the fungal cultures after the final time point of the growth curve in panel A. No growth was observed in the presence of NaD1, and the growth of *F. graminearum* in the presence of BPTI occurred in dense clumps, whereas growth in the presence of other PIs and the no-protein control was relatively homogeneous.

To assess whether the protease inhibitors had any effect on the growth rate of *F. graminearum* that had not been detected at the end time point used in the synergy assays, the growth of *F. graminearum* was evaluated over a 66-h time period in the presence of the PIs. Fungal growth (measured by the absorbance at 595 nm) in the presence of protease inhibitors (10 µM), NaD1 (2 µM), or H_2_O was measured every 30 min over the 66-h time course experiment (Fig. 3). *F. graminearum* did not grow in the presence of NaD1. BPTI inhibited growth for a short time period, but the fungus recovered rapidly from this inhibition. *F. graminearum* treated with BBI, LBTI, and CI-1B showed growth patterns similar to *F*. *graminearum* treated with the water control. There was a difference in the morphology of the fungus that grew in the presence of BPTI compared to fungus grown in the presence of the other PIs and H_2_O. In the presence of BPTI, *F. graminearum* formed dense bundles of hyphae, whereas a relatively homogeneous mat was formed in the presence of BBI, LBTI, or CI-1B, as occurred with the no-protein control.

### Effects of serine protease inhibitors on *C. albicans* stress response mutants.

**FIG 4:**
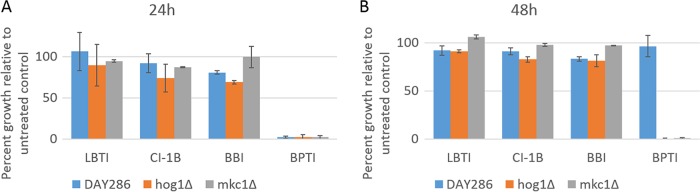
Activity of PIs against *C. albicans* signaling mutants and the wild-type strain. (A and B) Effects of protease inhibitors (20 µM) on the growth of wild-type *C. albicans* DAY286 and *hog1*Δ and *mkc1*Δ mutants displayed as percent growth of the untreated control strain after incubation for 24 and 48 h. BPTI is the only protease inhibitor that completely inhibited growth of all strains after 24 h (A). After 48-h growth, the wild-type DAY286 strain recovered to close to 100% of the untreated control, but the two stress response mutants did not recover, indicating that they have a defect in overcoming the growth inhibition induced by BPTI (B). Some growth inhibition was detected in DAY286 strain and the *hog1*Δ strain treated with BBI, but not for the *mkc1*Δ strain.

Fungi protect themselves against stress through activation of MAP kinase signaling cascades that result in the expression of genes that function to counter the effects of a specific stressor. For example, the high-osmolarity glycerol (HOG) pathway is activated in the fungal response to the plant defensin NaD1 and enables the fungus to survive at low NaD1 concentrations (26). Thus, we assessed whether interference or overloading of stress response pathways by the PIs was the cause of synergy rather than direct inhibition of a crucial fungal protease. *C. albicans* strains with deletions in key components of the Hog1 pathway (*hog1*Δ) and the cell wall integrity pathway (*mkc1*Δ) and the isogenic wild-type strain DAY286 were treated with 20 µM concentrations of BPTI, LBTI, BBI, and CI-1B. The impact of the PIs on fungal growth was assessed by measuring the optical density after 24 and 48 h of incubation. At 24 h, BPTI inhibited growth of the wild-type strain and both deletion strains. At 48 h, growth of the wild-type DAY286 strain had recovered, but the *hog1*Δ and *mkc1*Δ strains were still inhibited. LBTI, BBI, and CI-1B did not significantly inhibit any of the strains tested at either time point (Fig. 4).

### Synergy between BPTI and other antifungal molecules.

BPTI had the strongest synergy of the PIs tested with NaD1. To determine whether this was specific to NaD1 or whether BPTI could enhance the activity of other molecules, we assessed the synergy between BPTI and a variety of other antifungals against *C. albicans* (Table 6). The combination of BPTI and the echinocandin caspofungin or the plant defensin DmAMP1 (*Dahlia merckii* antimicrobial peptide 1) both had FIC scores of less than 0.5, indicating a synergistic interaction. The FIC values for BPTI with the two membrane-permeabilizing peptides LL37 and CP29, the plant defensin NaD2, the human beta defensin HBD2 and the histatin HST5 were all between 0.5 and 1, indicating that there was not a strong synergistic interaction. When BPTI was used in combination with the ergosterol synthesis inhibitor fluconazole, the FIC value was 1, indicating that there was no interaction between the two antifungals.

**TABLE 6:** Synergy between BPTI and other antifungal molecules against *C. albicans*

Molecule	FIC^a^
Caspofungin	0.27 (0.10)
CP29	0.54 (0.07)
DmAMP1	0.19 (0.00)
Fluconazole	1.00 (0.00)
HbD2	0.71 (0.31)
HST5	0.67 (0.07)
LL37	0.49 (0.16)
NaD2	0.69 (0.11)

a Data are the averages from three independent experiments. The standard deviations of the values from three experiments are presented in parentheses.

## DISCUSSION

The use of combinations of antimicrobial molecules that act synergistically against pathogens has been proposed as a method to improve their efficacy and prevent the development of resistance. Until now, most of the research has focused on synergy between families of molecules with reasonably well-defined and different mechanisms of action. Here we have identified a synergistic antifungal activity between the plant defensin NaD1, for which we understand some of the components of the mechanism of action, and a range of serine protease inhibitors which, apart from BPTI, have no antifungal activity. Synergy was assessed using two agricultural fungal pathogens, *F. graminearum* and *C. graminicola*, as well as the medically relevant fungal pathogen *C. albicans*. The only combination that met the initial criterion for synergy (FIC of <0.5) was NaD1 with BPTI against *C. albicans*. Determining synergy using the FIC calculation was complicated by the lack of growth inhibition and therefore lack of a measurable MIC for most of the protease inhibitors against the fungal species tested (BPTI inhibits *C. albicans* above the concentrations tested in the synergy assays) and required us to arbitrarily set the MIC at double the top concentration in our assay. Although the FIC values for our assays did not identify a synergistic interaction between NaD1 and PIs, we noticed that NaD1 was inhibiting fungal growth at substantially lower concentrations when PIs were added. This led to the use of an alternative method for determining synergy. Limpel’s formula calculates synergy based on the difference between the observed inhibition of fungal growth when two potential antifungal molecules are used in combination compared to the expected inhibition if the two molecules are tested separately and their combined activity is assumed to be additive. The greater the difference in inhibition (ID), the greater the synergy. A strong synergistic interaction determined using Limpel’s formula is defined as an ID greater than 50. The combination of BPTI with NaD1 had the highest ID for all fungal species tested. The other protease inhibitors had variable ID values when used in combination with NaD1 across the different fungal species. *F. graminearum* was the most susceptible to synergy with three of the four initial PIs tested resulting in an ID greater than 50 when used in combination with NaD1.

*In vitro* synergy between PIs and a plant defensin led to the idea that perhaps PIs could act as cofactors in the plant innate immune system. This led to a search for plant PIs that were upregulated in the response to fungal infection. At2g38870 is overexpressed in *Arabidopsis thaliana* plants that are more resistant to infection by *Botrytis cinerea* (27). When combined with NaD1, At2g38870 had a strong synergistic activity against *F. graminearum* growing *in vitro*. This may reflect the function of At2g38870 *in planta* as a peptide that facilitates fungal cell killing by improving the activity of endogenous *A. thaliana* defense proteins. This potential *in planta* role of At2g38870 should be tested further to determine whether this is the primary function of this PI.

The observation that a range of serine PIs acted synergistically with NaD1 initially led to the assumption that the synergy resulted from inhibition of fungal proteases in the extracellular space that were required for fungal viability. Alternatively, the defensin may have made the membrane more permeable to small peptides, thus allowing access to intracellular protease targets. However, the lack of synergy with small-molecule protease inhibitors and the persistence of synergy with BPTI and CI-1B that had been inactivated by substitution of their reactive-site residues indicate that proteases are unlikely to be the target of the protease inhibitors in the synergistic interaction with NaD1. Furthermore, no correlation was found between the inhibition of trypsin or chymotrypsin and the level of synergy observed for each PI. To further the argument that protease inhibition is not contributing to synergy with NaD1, BPTI also had strong synergistic activity with caspofungin, which is not a protein and would therefore not be affected by a protease and would thus not be expected to act synergistically with a molecule that blocked the activity of proteases. BPTI inhibits fungal growth by preventing uptake of Mg^2+^ and not via inhibition of fungal proteases (25). The other protease inhibitors may not be targeting fungal proteases and may act by affecting other processes that increase the susceptibility of the fungus to NaD1.

Although *F. graminearum* was susceptible to synergy between NaD1 and most PIs, we did not detect any effect of the PIs alone on the growth of the fungus at the 48-h endpoint. NaD1 can act on fungal cells within 30 min (26) so we considered whether the PIs were affecting the rate of growth of the fungus and whether this was having an effect on the activity of NaD1. BPTI was the only PI that had any effect on the growth of *F. graminearum*. The increased lag phase could result in inhibition of growth by lower concentrations of NaD1. Slowing the growth of the fungus would have the same effect as decreasing the starting cell density. That is, retardation of growth by BPTI swings the tug-of-war between cell killing and cell growth to favor cell killing by NaD1, or synergy. However, this does not explain the increased activity of NaD1 in the presence of the other PIs.

With the goal of further elucidating the mechanism of synergy between NaD1 and PIs, we turned to what is known about the antifungal mechanism of NaD1. A stress response MAP kinase (MAPK) cascade functions in the fungal response to subinhibitory levels of NaD1. We hypothesized that PIs have an effect on stress response pathways that affect the ability of the fungus to respond to NaD1. The inability of the *hog1*Δ and *mkc1*Δ strains to recover from BPTI-induced growth arrest suggests that when these pathways are activated, they protect the cells from damage by low concentrations of BPTI. Synergy could result from overloading of these stress response pathways when the cell is exposed to both NaD1 and BPTI, thus enhancing sensitivity to both molecules.

The sequence diversity and lack of consistency at producing a synergistic effect with NaD1 across the limited number of fungal species tested indicate that the mechanism of synergy with each PI is different. Some insight has been provided on potential mechanisms of synergy between NaD1 and BPTI through experimentation, but the exact mechanism is still unclear. One feature that separates BPTI from the other three PIs is that BPTI is the only molecule that has a positive charge at pH 7. CI-1B, BBI, and LBTI are all negatively charged proteins. Perhaps there is an interaction between these PIs and the positively charged NaD1 that facilitates transit of the defensin through the cell walls of specific fungal species. Alternatively, these PIs could each have very different effects on the fungal cell that will require transcriptomic or proteomic analysis to identify.

To determine whether the synergistic activity of NaD1 and BPTI was specific to NaD1, we also assessed a selection of other antifungals for synergy with BPTI. Synergy was detected between BPTI and the 1,3-β-glucan synthase inhibitor caspofungin (28) and between BPTI and the plant defensin DmAMP1, which interacts with sphingolipids in the fungal cell wall (24). Although the cutoff for synergy of a FIC of 0.5 was obtained only with the combination of BPTI with caspofungin or DmAMP1, BPTI did increase the activity of many of the other antifungals assessed. This indicates that there is a subset of molecules for which BPTI can act synergistically even though the underlying mechanism is still unclear. It does decrease the likelihood that synergy is due to protein-protein or protein–small-molecule interaction between BPTI and the synergy partner, as it is unlikely that a diverse set of molecules would all participate in similar intermolecular interactions. The only similarity in the mechanisms of caspofungin, DmAMP1, and NaD1 is that there is some involvement of the fungal cell wall. However, the relationship between the cell wall and each of these antifungals differs significantly. Perhaps BPTI gains entry to the cell to alter cell wall dynamics. Synergy is likely to be a complex, multifactorial process, which will require further investigation to truly understand.

Antimicrobial synergy is most often investigated for molecules that both have antimicrobial activity on their own. Here we report the synergy between an antifungal molecule, NaD1, and a molecule that has some antifungal activity, BPTI, as well as a set of molecules (LBTI, BBI, and CI-1B) that have no effect on fungal growth when used in isolation. These experiments have been conducted in controlled *in vitro* conditions, but we hypothesize that there are a multitude of plant peptides and small molecules in plant tissues that enhance the antifungal activity of plant defensins and other antifungal peptides *in planta* as with the PIs reported here. That is, there is likely to be a collection of molecules that serve as antimicrobial cofactors that increase the susceptibility of microbes to the antimicrobials produced by the plant. Like the PIs, they may not have been identified because they have no antifungal activity on their own. This could represent the next step in the evolutionary tug-of-war between plants and microbial pathogens, as developing resistance to the activities of two molecules is significantly more challenging for the microbe than developing resistance to one.

## MATERIALS AND METHODS

### Strains and culture conditions.

*Fusarium graminearum* (Fgr, PH-1) spores were isolated from fungi on synthetic nutrient-poor agar (0.1% KH_2_PO_4_, 0.1% KNO_3_, 0.1% MgSO_4 _⋅ 7H_2_O, 0.05% KCl, 0.02% glucose, 0.02% sucrose, 2% Bacto agar). *Colletotrichum graminicola* (US isolate Carroll-1A-99) spores were isolated from fungi on clarified V8 agar. Culturing of filamentous fungi was performed in 1/2 strength potato dextrose broth (1/2 PDB) at room temperature. *Candida albicans hog1*Δ/*hog1*Δ and *mkc1*Δ/*mkc1*Δ deletion strains were retrieved from the *C. albicans* transcription factor deletion collection (29). These strains, the isogenic wild-type DAY286 strain and ATCC 90028, were maintained on YPD agar plates (2% peptone, 1% yeast extract, 2% glucose, 2% Bacto agar) and grown in liquid culture in YPD.

### Expression and purification of proteins.

The plant defensins NaD1 and NaD2 were extracted from flowers of *Nicotiana alata* as described previously (30). DmAMP1 (*Dahlia merckii* antimicrobial peptide 1) (31) and HbD2 (32) were expressed in *Pichia pastoris* and purified using ion-exchange chromatography and reverse-phase high-performance liquid chromatography (RP-HPLC) as described previously (26). An inactive variant of bovine pancreatic trypsin inhibitor (BPTI) (25) (iBPTI with K15A and R17A substitutions), wild-type barley chymotrypsin inhibitor CI-1B (33), an inactive variant of CI-1B (iCI-1B with L63A substitution), At2g38870, a PR6-like protein with serine protease inhibitory activity from *Arabidopsis thaliana* (GenBank accession no. BT005182), NaPin1a, a serine protease inhibitor from *N. alata* (GenBank accession no. KY436037), and NaCys1, a cysteine protease inhibitor from *N. alata* (GenBank accession no. KY436036) were all expressed using the pHUE system (34) in *Escherichia coli* Rosetta-gami (Merck). The correct mass for all expressed and purified protease inhibitors (PIs) was confirmed by matrix-assisted laser desorption ionization–time of flight (MALDI-TOF) mass spectrometry using a Bruker Ultraflex III TOF/TOF mass spectrometer. BPTI was purchased from Amresco as high-purity aprotinin. Lima bean trypsin inhibitor (LBTI) and soybean trypsin inhibitor were purchased from Sigma (as trypsin inhibitor type II-L; lima bean and trypsin-chymotrypsin inhibitor from *Glycine max* [soybean], respectively). The human cathelicidin LL37 (35) and the cercropin-melitin hybrid CP-29 (36) were synthesized by GL Biochem (Shanghai, China). Histatin 5 (37) was purchased from mimotopes (Clayton, Australia). Small-molecule protease inhibitors, caspofungin, and nikkomycin were all purchased from Sigma.

### Synergy assays.

Inhibition of fungal growth was assessed based on the protocol described in reference 38. *F. graminearum* or *C. graminicola* spores were isolated by flooding cultures on agar plates with sterile H_2_O, counted using a hemocytometer, and diluted to 5 × 10^4^ spores/ml in 1/2 PDB. *C. albicans* was cultured in liquid YPD overnight at 30°C, cells were counted using a hemocytometer, and diluted to 5 × 10^3^ cells/ml. Defensins, protease inhibitors and other molecules to be tested in synergy assays were prepared at 10 times the desired concentration. Aliquots of the test molecules (10 µl) were arrayed into the wells of a 96-well microtiter plate using a Tecan Freedom EVO liquid-handling robot or by hand. Spores or cells (80 µl) were then added to all the wells of the plate and incubated at 25°C for 48 h (*F. graminearum* or *C. graminicola*) or 30°C for 24 h (*C. albicans*). Growth was monitored by measuring the optical density at 595 nm using a SpectraMax M5e plate reader (Molecular Devices). Synergistic interactions were identified using the checkerboard method and a fractional inhibitory concentration (FIC) (39) cutoff of 0.5 (FIC = MIC_A_combination/MIC_A_alone + MIC_B_combination/MIC_B_alone where MIC_A_combination is the MIC of agent A in combination and MIC_A_alone is the MIC of agent A alone) as described previously (28). When inhibition was not observed for a particular molecule when used in isolation, the MIC for that molecule was set to two times the top concentration tested. Limpel’s formula [Ee = *X* + *Y* − (*XY*)/100, where *X* and *Y* represent the percentage inhibition obtained from the individual components *X* and *Y*] was used as a second test for synergistic interactions (40), as there were issues with the FIC test when using molecules with no measurable MIC. Ee is the degree of inhibition expected if the individual components do not interact. Io is the observed inhibition for the combination of molecules. To assess the extent of the synergistic interaction by this method, we have used the term inhibition difference (ID). Inhibition difference is calculated by subtracting the expected inhibition (Ie) from the observed inhibition (Io) and is therefore the additional growth inhibition achieved when using the compounds together compared to the predicted inhibition based on their activities alone. Synergy is identified when the inhibition difference (ID) (Io – Ie) is greater than zero and is highest as ID approaches 100. There is no synergy when ID is zero, and there is antagonism when ID is less than zero.

### Protease inhibition.

Bovine chymotrypsin (treated with *N*α-*p*-tosyl-l-lysine chloromethyl ketone [TLCK]) and trypsin were purchased from Sigma. Active site titration of the enzyme stocks was performed using 4-methylumbelliferyl 4-guanidinobenzoate (MUGB) and 4-methylumbelliferyl *p*-trimethylammoniocinnamate (MUTMAC) for trypsin and chymotrypsin, respectively (41). The stock of the titrant MUTMAC was prepared as described previously (41), while MUGB was prepared in dimethyl sulfoxide (DMSO).

The inhibitor stocks were titrated using titrated trypsin, and the residual activity was assessed assuming a 1:1 interaction between enzyme and inhibitor (42). Assays were performed in buffer (50 mM Tris-HCl [pH 8.0], 150 mM NaCl, 0.01% Triton X-100) by incubating the enzyme (25 nM) with various concentrations of inhibitor (0 to 100 nM). The mixture was allowed to preincubate for 15 min before addition of the substrate *N*-α-benzoyl-dl-Arg-*p*-nitroanilide.

When the inhibitors could not be titrated using the enzyme (i.e., when the affinity was too poor), their concentrations were estimated using absorbance at 215 nm and a theoretical molar extinction coefficient. Theoretical extinction coefficients were estimated according to the algorithm described by Moffatt et al. (43).

The titrated enzyme was incubated at room temperature (RT) with different concentrations of inhibitor (0 to 1 µM). Variable incubation times (60 or 240 min) were used to assess equilibrium of the enzyme-inhibitor complex. The buffers used for the inhibition assays were 50 mM Tris-HCl, pH 8.0, 150 mM NaCl, and 0.01% Triton X-100 (for trypsin) and 80 mM Tris-HCl, pH 7.8, 10 mM Ca_2_Cl, and 0.01% Tween 20 (for chymotrypsin). The assay plates with the enzymes and inhibitors were preincubated for 30 min at 37°C, followed by the addition of substrate (*t*-butyloxycarbonyl-Gln-Ala-Arg-7-amino-4-methylcoumarin and *N*-succinyl-Ala-Ala-Pro-Phe-7-amido-4-methylcoumarin for trypsin and chymotrypsin, respectively), and then the absorbance in each well was measured using a SpectraMax M5e plate reader in kinetic mode at 37°C. The resulting data were fitted to Morrison’s equation (44) using GraphPad Prism V5.01 for Windows. The values presented are mean values ± standard errors of the means (SEM) where *n* ≥ 3.

### Fungal growth curves.

*F. graminearum* spores were diluted to 5 × 10^4^ spores/ml in 1/2 PDB. Protease inhibitors were all prepared at 100 µM, and NaD1 was prepared at 20 µM in MilliQ H_2_O. Ten microliters of each protease inhibitor or defensin was transferred to one column of 8 wells in a 96-well plate. A control column with MilliQ H_2_O in place of the test protein was also prepared. *F. graminearum* spore suspension in 1/2 PDB (90 µl) was added to the wells of the 96-well plate, and the absorbance at 595 nm was measured every 30 min for 66 h. Absorbance at 595 nm for each time point was averaged across the eight replicates and plotted against time. Data presented are representative of the three biological replicates.

### Antifungal assays.

Growth inhibition assays on *C. albicans* deletion strains and the corresponding wild-type strain were performed as described previously (26).

### Accession number(s).

Sequence accession data for NaPin1a (KY436037) and NaCys1 (KY436036) have been deposited in GenBank.
